# miR-155 is involved in Alzheimer’s disease by regulating T lymphocyte function

**DOI:** 10.3389/fnagi.2015.00061

**Published:** 2015-04-30

**Authors:** Juhyun Song, Jong Eun Lee

**Affiliations:** ^1^Department of Anatomy, Yonsei University College of MedicineSeoul, South Korea; ^2^Brain Korea 21 Plus Project for Medical Science and Brain Research Institute, Yonsei University College of MedicineSeoul, South Korea

**Keywords:** micro RNA-155 (miR-155), Alzheimer’s disease (AD), T lymphocyte (T-cell), immunity

## Abstract

Alzheimer’s disease (AD) is considered the most common cause of sporadic dementia. In AD, adaptive and innate immune responses play a crucial role in clearance of amyloid beta and maintenance of cognitive functions. In addition to other changes in the immune system, AD alters the T-cell responses that affect activation of glial cells, neuronal cells, macrophages, and secretion of pro-inflammatory cytokines. These changes in the immune system influence AD pathogenesis. Micro-RNA (miRNA)-155 is a multifunctional miRNA with a distinct expression profile. It is involved in diverse physiological and pathological mechanisms, such as immunity and inflammation. Recent studies indicate that miR-155 regulates T-cell functions during inflammation. In this article, we summarize recent studies describing the therapeutic potential of miR-155 via regulation of T cells in AD. Further, we propose that regulation of miR-155 might be a new protective approach against AD pathogenesis.

## Introduction

Alzheimer’s disease (AD) is a major cause of dementia in humans, and about 27 million people suffer from this disorder (Wimo et al., 2006; Rubio-Perez and Morillas-Ruiz, 2012). Neuroinflammation, a pathological hallmark of AD, occurs in susceptible regions in the AD brain (Griffin and Mrak, 2002; Cacquevel et al., 2004; Finch and Morgan, 2007; Di Bona et al., 2008, 2009; Rubio-Perez and Morillas-Ruiz, 2012) and plays an important role in AD progression (Ke et al., 2006). During AD, neuroinflammation increases the concentrations of pro-inflammatory cytokines (Bauer et al., 1991; Strauss et al., 1992; Remarque et al., 2001) and percentage of activated immune cells (Lombardi et al., 1999; Speciale et al., 2007; Saresella et al., 2011). Furthermore, it regulates accumulation of inflammatory molecules and activated glial cells in the surroundings of amyloid plaques in the brain of patients with AD and animal models (Bauer et al., 1991; Fillit et al., 1991; Cagnin et al., 2001). However, the functions of both the inflammatory and immune components need to be further investigated in AD pathogenesis (Lal and Forster, 1988; McGeer and McGeer, 2002; Steinman, 2006). Adaptive immune cells such as T and B lymphocytes play important roles in inflammatory responses in the AD brain. Several studies report that differentiation of cluster of differentiation (CD) 3+ T-cells in AD hippocampal parenchyma is increased compared with controls (Togo et al., 2002) and that T-cells are activated and infiltrate into the AD brain. In addition, subsets of T-cells in blood circulation as well as in the brain parenchyma are altered in AD (Town et al., 2005). During infiltration, T cells produce interferon gamma (IFN-γ) that leads to the deposition of amyloid beta peptides (Aβ) and subsequently, cognitive dysfunction (Browne et al., 2013). MicroRNAs (miRNAs) are single-stranded, ~22 nucleotide long non-coding RNAs that regulate gene expression (Kim and Nam, 2006). Several miRNAs are expressed in the brain and are involved in inflammation and microglia activation (Faraoni et al., 2009; Junker et al., 2009; Buck et al., 2010), cell cycle regulation (Johnson et al., 2007; Schultz et al., 2008) and in apoptosis (Chhabra et al., 2009). Recent studies report that miRNAs are also associated with the T cell functions, such as T cell activation and development (Gatto et al., 2008; Rusca et al., 2012; Yang et al., 2012). Among a number of miRNAs, miR-155 reportedly regulates inflammatory and immune responses via modulation of suppressor of cytokine signaling 1 (SOCS1; Dudda et al., 2013), activator protein 1 (Yin et al., 2008), and signal transducers and activators of transcription 5 (STAT5; Kopp et al., 2013). It is observed to be associated with multiple processes, such as regulation of IFN-γ signaling and thus, CD8+ T-cell activation (Gracias et al., 2013), T cell development (Kohlhaas et al., 2009; O’Connell et al., 2009), cell-cell interactions (Martin et al., 2006), and macrophage activation (O’Connell et al., 2007). Recent research demonstrates that the expression of several miRNAs change in AD (Nelson and Keller, 2007; Nelson et al., 2008; Barbato et al., 2009; Kocerha et al., 2009); including in the brain tissue and cerebrospinal fluid (Cogswell et al., 2008). Accordingly, miR-155 expression has been observed to be altered in brain tissue from patients with AD (Culpan et al., 2011). It enhances neuroinflammation in AD progression in a triple transgenic mouse model of AD (Guedes et al., 2014). In this review, we present a new perspective regarding the regulatory role of miR-155 in T-cell functions and thus, AD progression.

## T-Cell Response in AD

In AD, interaction between the central nervous system and immune system is facilitated by lymphocytes in the blood and by immune mediators (Britschgi and Wyss-Coray, 2007). During an inflammatory response, immune cells in the blood migrate and infiltrate the brain. However, the level of T-cells in the brain is significantly lower in AD than in other neurodegenerative diseases, such a multiple sclerosis or Parkinson’s disease (Lafaille, 1998; Nagelkerken, 1998). In normal, unaffected patients, there are few T-cells in the brain; however, due to disruption of the blood brain barrier (BBB), this number increases in the AD brain, specifically in the hippocampus and temporal cortex (Sardi et al., 2011). T-cells are derived from lymphoid stem cells in the bone marrow and mature in the thymus (Starr et al., 2003). Based on the expression of surface molecules such as CD3, CD4, and CD8, the development of T-cells in the thymus has been divided into three stages: initial, intermediate, and final (Starr et al., 2003). Mature T-cells are classified into naïve, effector, and memory T-cells. Each subset expresses specific surface molecules, such as the C-C chemokine receptor type 7 (CCR7), CD45RA, CD70, and CD27 (Romero et al., 2007; Salaun et al., 2011). Based on cytokine profiles, T helper (Th) cells are divided into Th1, Th2, Th9, and Th17 cells depending on the activity of other immune cells and based upon their ability to produce various cytokines (Harrington et al., 2005; Baumjohann and Ansel, 2013). A study of immune parameters in AD reports a decrease in the percentage of naïve T-cells, an increase in the number of memory T-cells and CD4+ T-cells, and a reduction of regulatory T-cells (Tregs) compared with the control group (Larbi et al., 2009). Furthermore, a clinical study of AD reports a significant reduction of naïve CD4+ T-cells in these patients and an increase in number of late-differentiated memory T-cells (Pellicano et al., 2012). Xue and colleagues report a significant reduction of CD3+ T-cells, but marginal changes in CD4+ and CD8+ T-cell subsets in AD (Xue et al., 2009). Richartz-Salzburger and colleagues confirmed the decrease of CD3+ and CD8+ T-cell number, but showed a minor increase in CD4+ cells in AD (Richartz-Salzburger et al., 2007). Several studies report that CD45RO+ T-cell expression increases in the brains of patients with AD (Togo et al., 2002; Lombardi et al., 2004). Further, Lombardi and colleagues (Lombardi et al., 1999) showed an increase in the CD4+ Th and CD25+ Treg subsets in patients with AD. Other studies report that CD45RO+ T-cell expression increases in the amyloid-beta peptide (Aβ), a marker of AD, has been reported to stimulate the macrophage inflammatory protein (MIP)-1α expression in peripheral T-cells and its receptor C-C chemokine receptor type 5 (CCR5) expression in brain endothelial cells. These alterations in signaling help T-cells cross the BBB (Man et al., 2007). In addition, accumulation of Aβ in AD stimulates microglia, which secrete granulocyte macrophage-colony stimulating factor (GM-CSF) to regulate antigen presentation (Tarkowski et al., 2001). Furthermore, circulating Aβ-reactive T-cells are observed in patients with AD (Monsonego et al., 2003). Interestingly, animal studies using APP/PS1 mice demonstrate that Aβ-reactive Th1 cells stimulate microglial activation (Browne et al., 2013) and decrease Aβ pathology (Butovsky et al., 2006; Ethell et al., 2006; Monsonego et al., 2006; Fisher et al., 2010). By secreting Th2-type cytokines (downregulate proinflammatory responses), Aβ-reactive T-cells reduce development of AD symptoms (Weiner et al., 2000; Tarkowski et al., 2001). Additionally, astrocytes secrete transforming growth factor–beta (TGF-β) that promotes Th2 responses and thus, alleviates Aβ pathology in an AD animal model (Wyss-Coray et al., 2001). Interestingly, a co-culture (T-cell and microglia) study demonstrates that Th1 cells up-regulate expression of major histocompatibility complex (MHC) class II and CD40, markers of antigen-presenting cells in microglia (Aloisi et al., 2000). Aβ-reactive Th1 cells increase the secretion of inflammatory cytokines such as interleukin (IL)-1β, IL-6, and tumor necrosis factor–alpha (TNF-α) and promote the expression of MHCII and CD86 in microglia (McQuillan et al., 2010). Th1 and Th17 cells increase microglial production of inflammatory cytokines and expression of MHCII, CD80, and CD86 (Murphy et al., 2010). In addition, hyperpermeability of the BBB in AD increases the infiltration of circulating immune cells, such as T-cells (Togo et al., 2002; Schindowski et al., 2007). In patients with AD, T-cell migration into the brain is followed by enhanced expression of MHC I and II in activated microglia (Mattila et al., 1994; Vugler et al., 2007). In AD, T-cells also participate in various activities, such as expression of neurotrophic factors (Aharoni et al., 2005; Butovsky et al., 2006; Hohlfeld et al., 2006) and neurogenesis (Butovsky et al., 2006; Baron et al., 2008; Mastrangelo et al., 2009; Wolf et al., 2009). Taken together, we propose that T-cells are one of the key regulators of pathological processes in AD. Thus, control of these cells may provide an effective treatment strategy for alleviating the pathogenesis of AD.

## MicroRNA

miRNAs are short, approximately 22 nucleotide-long, non-coding RNAs (Bartel, 2004) that regulate gene expression by stimulating either mRNA degradation or their translational repression by binding to the 3′-untranslated region of target mRNAs (Bartel et al., 2004; Bagga et al., 2005; Filipowicz et al., 2005; Chen et al., 2006). Similar to pre-mRNAs, a pri-miRNA sequence contains a CAP structure and ploy-A tail. Pri-miRNAs are transcribed by both RNA polymerase I and II (Lee et al., 2004). miRNAs play important roles in diverse mechanisms including cell proliferation, development, and differentiation (Gregory and Shiekhattar, 2005). They are not restricted to the cytoplasm, and are also functional in the nucleus (Foldes-Papp et al., 2009; Park et al., 2010). In humans, over 2500 miRNAs have been identified (Acunzo et al., 2014), and most are located at chromosomal regions exhibiting amplification, deletion, or translocation in various diseases, including cancer (Calin et al., 2004; Lu et al., 2005; Volinia et al., 2006), leukemia (Calin et al., 2004; Lawrie et al., 2007; Xu and Li, 2007), diabetes (Yu et al., 2014), cardiovascular disease (Maegdefessel, 2014), and AD (Cacabelos and Torrellas, 2014; Galimberti et al., 2014). Interestingly, miRNAs are also involved in the regulation of T-cell development, maturation, differentiation, and function (Neilson et al., 2007; Jindra et al., 2010). T-cells play an important role in the adaptive immune response. miR-155 is involved in multiple processes (Gatto et al., 2008; O’Connell et al., 2009), including inflammation (Tili et al., 2007), immunity (Kohlhaas et al., 2009; Sonkoly et al., 2010; Gracias et al., 2013; Kopp et al., 2013) and regulatory mechanisms in numerous diseases. The present review therefore emphasizes the role of miR-155 in T-cell alterations during AD pathology.

## miR-155 and T-Cell Responses in AD

Several studies report that the expression of miR-155, mediated by Toll-like receptors, increases in monocytic cell lines during lipopolysaccharide (LPS)-induced inflammation (Taganov et al., 2006; O’Connell et al., 2007). miR was shown to regulate acute inflammation after pathogen recognition by Toll-like receptors on monocytes or macrophages; thus, it was involved in innate immunity (Taganov et al., 2006; O’Connell et al., 2007). In addition, inflammatory cytokines such as IFN-α, γ, and TNF-α also strongly stimulate miR-155 expression. These findings indicate that miR-155 is a component of the innate immune response that depends on functions of numerous inflammatory mediators (O’Connell et al., 2007). Interestingly, miR-155-null mice exhibit reduced IL-2 and IFN-γ production, indicating that it is necessary for T-cell responses (Rodriguez et al., 2007). In recent *in vivo* studies, elevated levels of miR-155 were observed following T-cell stimulation through the T-cell receptor (TCR; Thai et al., 2007; Dudda et al., 2013; Gracias et al., 2013). miR-155 is also required for development and generation of T cells after TCR activation *in vivo* (Georgantas et al., 2007), and also for T-cell response, such as dendritic cell-T-cell interactions (Tili et al., 2007; O’Connell et al., 2010). miR-155-deficient mice exhibit impaired antigen-presentation by dendritic cells as wells as defective dendritic cell-T-cell interactions (Rodriguez et al., 2007). Consequently, miR-155-null mice lack adequately activated T-cells (Rodriguez et al., 2007).

Further, miR-155 regulates BBB permeability in central nervous system neuroinflammatory disorders by regulating cell-cell interaction molecules in mouse brains (Lopez-Ramirez et al., 2014). As discussed above, miR-155 is associated with T-cell functions by regulating the TCR and inflammatory cytokine production. These evidence suggest that miR-155 is involved in T-cell immune functions and thus, in the inflammation during AD. Therefore, we summarize the multiple roles of miR-155 in functions of different T-cell types.

## Th1, Th2 and Th17 Cells

**Figure 1:**
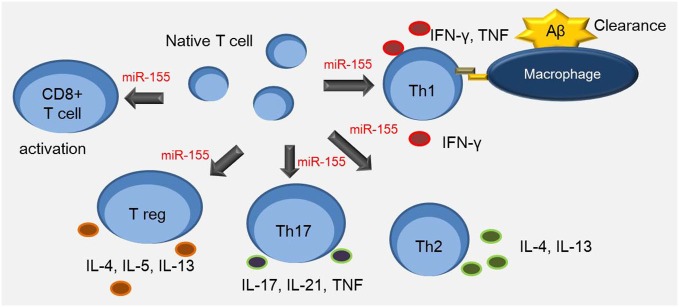
**miR-155 is involved in the T cell response**. Th1 cells up-regulate expression of major histocompatibility complex (MHC) class II and CD86 in antigen presenting cells such as macrophages. Aβ-reactive Th1 cells increase the secretion of inflammatory cytokines such as IFN-γ and TNF-α. miR-155 is associated with multiple process including the interaction between dendritic cells and T cells, and the regulation of Th17 and CD4+ T cell differentiation. It is also involved in regulating proliferation of Th1, Th2, and CD8+ T cells, and survival of Treg cells.

**Figure 2:**
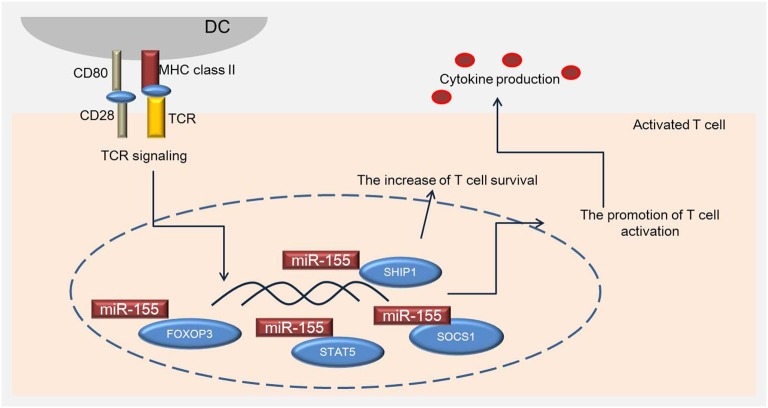
**miR-155 is associated with specific transcription genes regulating activation of T cells**. miR-155 regulates the development of Treg cells by inducing FOXP3, which plays an important role in Treg cell survival *in vivo*, and regulates the phosphorylation of STAT5 and SOCS1. SHIP1 increases the survival of T cells by modulating IFN-γ production.

Recent *in vitro* studies report that the expression of miR-155 is up-regulated in activated T-cells (Tam, 2001; Cobb et al., 2006). Thai et al. observed that miR-155-deficient mice have reduced germinal center function, T-cell dependent immune responses, and cytokine production (Thai et al., 2007). In addition, the immune responses in miR-155-deficient mice are diverted toward a Th2 pattern, with a significant increase of IL-10, which mediates immunosuppressive effects against cell-mediated responses (Thai et al., 2007). In addition, T-cells from miR-155-null mice show an increased tendency to differentiate into Th2 type cells; they enhanced Th2-type cytokine production when cultured *in vitro* (Rodriguez et al., 2007). On the other hand, elevated levels of miR-155 in activated CD4+ T-cells induce Th1 cell differentiation by targeting the IFN-γ receptor alpha chain (Banerjee et al., 2010), and miR-155 deficient CD4+ T-cells are more likely to polarize toward Th2 cells (Rodriguez et al., 2007; Banerjee et al., 2010). miR-155 specifically targets c-Maf, affecting activation of Th2 specific cytokine IL-4 (Rodriguez et al., 2007). A reduced number of IFN-γ-producing cells lacking miR-155 results in T-cell dysfunction and antigen-presentation defects (O’Connell et al., 2009). Phosphatidylinositol 3, 4, 5-trisphosphate 5-phosphatase 1 (SHIP1) has also been suggested as a functional target of miR-155 in CD4+ T cells e.g., macrophages (O’Connell et al., 2009) and dendritic cells (O’Connell et al., 2010). The levels of SHIP1 are reduced in miR-155^−/−^ mice. SHIP1 suppresses Th1 responses (Tarasenko et al., 2007) and T-cells by modulating IFN-γ production (Huffaker et al., 2012). In human CD4+ T-cells, miR-155 targets the IFN-γ receptor alpha subunit and regulates proliferation of the Th1 and Th2 subsets (Banerjee et al., 2010). Th17 cells are a newly defined subset of CD4+ T-cells that modulate autoimmunity by producing pro-inflammatory cytokines, including IL-17, IL-21, and IL-22 (Langrish et al., 2005; Korn et al., 2007; Miossec et al., 2009). miR-155-deficient mice are characterized by reduced numbers of Th17 cells, and thus, suggest that miR-155 is required for Th17 differentiation (O’Connell et al., 2010; Figures 1, 2). Taken together, miR-155 appears to regulate the differentiation, proliferation, and activation of Th1, Th2, and Th17 cells in the inflammatory state.

## Treg Cells

Treg cells play an important role in regulating the immune response and preventing autoimmunity (Tang and Bluestone, 2006). Both mouse and human Treg cells express a set of miRNAs (Cobb et al., 2006; Rouas et al., 2009; Smigielska-Czepiel et al., 2014). miR-155 has been reported to regulate the development of Treg cells by inducing forkhead box P3 (Foxp3), which regulates Treg cell survival *in vivo* (Kohlhaas et al., 2009; Lu et al., 2009). In line with this finding, miR-155 knock-out mice are observed to have reduced Treg cell numbers. Consequently, they had reduced STAT5 phosphorylation and IL-2 receptor signaling due to insufficient SOCS1 suppression (Lu et al., 2009). Other studies postulate that miR-155 deficiency results in reduced numbers of Treg cells due to decreased proliferation and increased apoptosis (Lu et al., 2010; Skinner et al., 2014; Figures 1, 2). Nevertheless, miR-155 appears to modulate the activation and proliferation of Treg cells during inflammation. The evidence suggests that miR-155 also regulates the Treg cell-mediated inflammation during AD.

## CD8+ T-Cells

Differentiation of naïve CD8+ T-cells into effector or memory cytotoxic T-cells (CTLs) depends upon activation following interaction with antigen-presenting cells (Zhang and Bevan, 2010). A deficiency of miR-155 decreases CD8+ T-cell responses, whereas miR-155 overexpression increases CD8+ T-cell responses during inflammation (Dudda et al., 2013; Gracias et al., 2013; Lind et al., 2013). Antigen-specific CD8+ T cells lacking miR-155 show increased phosphorylation of STAT1 in response to Type I interferon signaling (Gracias et al., 2013). Inhibition of STAT1 and interferon regulatory factor 7 (IRF7) partially ameliorates the immune dysfunction of miR-155 deficient CD8+ T-cells *in vivo* (Gracias et al., 2013). Dudda et al. report that miR-155 deficient CD8+ T-cells exhibit improved immune systems following SOCS1 overexpression (Dudda et al., 2013). Taken together, miR-155 appears to affect the activation of CD8+ T-cells, which are involved in the expression of STAT1, IRF7, and SOCS1 during inflammation.

## Conclusions

Inflammatory and immune responses play a crucial role in AD pathogenesis. Thus, an appropriate regulation of diverse T-cell types may alleviate AD related severe pathologies. miR-155 controls characteristics such as survival, differentiation, proliferation, and activation of Th1, Th2, Th17, Treg, and CD8+ T-cells during inflammation. Admittedly, miR-155 is not easy to identify the absolute beneficial function or the absolute negative function on inflammation caused in AD through T cell regulation, suggesting that it is associated with the various T cell type responses and the complicated T cell signaling. However, this review suggests promising approaches for AD treatment, involving control of miR-155. Although findings from clinical studies are still in the preliminary stages, further studies involving modulation of miR-155 levels could enable development of effective treatments for AD.

## Author Contributions

JS obtained the information and wrote the preliminary draft. JEL reviewed and revised the manuscript. JS and JEL revised details of the manuscript and provided overall supervision.

## Conflict of Interest Statement

The authors declare that the research was conducted in the absence of any commercial or financial relationships that could be construed as a potential conflict of interest.

## Abbreviations

miR-155: micro RNA-155
AD: Alzheimer’ disease
T-cell: T lymphocyte
BBB: blood brain barrier
CD: cluster of differentiation
Aβ: amyloid-beta peptide.

## References

[B1] Acunzo M. Romano G. Wernicke D. Croce C. M. (2014). MicroRNA and cancer—a brief overview. Adv. Biol. Regul. 57, 1–9. 10.1016/j.jbior.2015.02.001 25294678

[B2] Aharoni R. Arnon R. Eilam R. (2005). Neurogenesis and neuroprotection induced by peripheral immunomodulatory treatment of experimental autoimmune encephalomyelitis. J. Neurosci. 25, 8217–8228. 10.1523/jneurosci.1859-05.2005 16148229 PMC6725544

[B3] Aloisi F. De Simone R. Columba-Cabezas S. Penna G. Adorini L. (2000). Functional maturation of adult mouse resting microglia into an APC is promoted by granulocyte-macrophage colony-stimulating factor and interaction with Th1 cells. J. Immunol. 164, 1705–1712. 10.4049/jimmunol.164.4.1705 10657614

[B4] Bagga S. Bracht J. Hunter S. Massirer K. Holtz J. Eachus R.. (2005). Regulation by let-7 and lin-4 miRNAs results in target mRNA degradation. Cell 122, 553–563. 10.1016/j.cell.2005.07.031 16122423

[B5] Banerjee A. Schambach F. DeJong C. S. Hammond S. M. Reiner S. L. (2010). Micro-RNA-155 inhibits IFN-gamma signaling in CD4+ T cells. Eur. J. Immunol. 40, 225–231. 10.1002/eji.200939381 19877012 PMC2807623

[B6] Barbato C. Arisi I. Frizzo M. E. Brandi R. Da Sacco L. Masotti A. (2009). Computational challenges in miRNA target predictions: to be or not to be a true target? J. Biomed. Biotechnol. 2009:803069. 10.1155/2009/803069 19551154 PMC2699446

[B7] Baron R. Nemirovsky A. Harpaz I. Cohen H. Owens T. Monsonego A. (2008). IFN-gamma enhances neurogenesis in wild-type mice and in a mouse model of Alzheimer’s disease. FASEB J. 22, 2843–2852. 10.1096/fj.08-105866 18390924

[B8] Bartel D. P. (2004). MicroRNAs: genomics, biogenesis, mechanism and function. Cell 116, 281–297. 10.1016/S0092-8674(04)00045-5 14744438

[B9] Bartel F. Pinkert D. Fiedler W. Kappler M. Würl P. Schmidt H.. (2004). Expression of alternatively and aberrantly spliced transcripts of the MDM2 mRNA is not tumor-specific. Int. J. Oncol. 24, 143–151. 10.3892/ijo.24.1.143 14654951

[B10] Bauer J. Strauss S. Schreiter-Gasser U. Ganter U. Schlegel P. Witt I.. (1991). Interleukin-6 and alpha-2-macroglobulin indicate an acute-phase state in Alzheimer’s disease cortices. FEBS Lett. 285, 111–114. 10.1016/0014-5793(91)80737-n 1712317

[B11] Baumjohann D. Ansel K. M. (2013). MicroRNA-mediated regulation of T helper cell differentiation and plasticity. Nat. Rev. Immunol. 13, 666–678. 10.1038/nri3494 23907446 PMC3980848

[B12] Britschgi M. Wyss-Coray T. (2007). Systemic and acquired immune responses in Alzheimer’s disease. Int. Rev. Neurobiol. 82, 205–233. 10.1016/s0074-7742(07)82011-3 17678963

[B13] Browne T. C. McQuillan K. McManus R. M. O’Reilly J. A. Mills K. H. Lynch M. A. (2013). IFN-gamma production by amyloid beta-specific Th1 cells promotes microglial activation and increases plaque burden in a mouse model of Alzheimer’s disease. J. Immunol. 190, 2241–2251. 10.4049/jimmunol.1200947 23365075

[B14] Buck A. H. Perot J. Chisholm M. A. Kumar D. S. Tuddenham L. Cognat V.. (2010). Post-transcriptional regulation of miR-27 in murine cytomegalovirus infection. RNA 16, 307–315. 10.1261/rna.1819210 20047990 PMC2811660

[B15] Butovsky O. Koronyo-Hamaoui M. Kunis G. Ophir E. Landa G. Cohen H.. (2006). Glatiramer acetate fights against Alzheimer’s disease by inducing dendritic-like microglia expressing insulin-like growth factor 1. Proc. Natl. Acad. Sci. U S A 103, 11784–11789. 10.1073/pnas.0604681103 16864778 PMC1544247

[B16] Cacabelos R. Torrellas C. (2014). Epigenetic drug discovery for Alzheimer’s disease. Expert Opin. Drug Discov. 9, 1059–1086. 10.1517/17460441.2014.930124 24989365

[B17] Cacquevel M. Lebeurrier N. Chéenne S. Vivien D. (2004). Cytokines in neuroinflammation and Alzheimer’s disease. Curr. Drug Targets 5, 529–534. 10.2174/1389450043345308 15270199

[B18] Cagnin A. Brooks D. J. Kennedy A. M. Gunn R. N. Myers R. Turkheimer F. E.. (2001). *In-vivo* measurement of activated microglia in dementia. Lancet 358, 461–467. 10.1016/s0140-6736(01)05625-2 11513911

[B19] Calin G. A. Liu C. G. Sevignani C. Ferracin M. Felli N. Dumitru C. D.. (2004). MicroRNA profiling reveals distinct signatures in B cell chronic lymphocytic leukemias. Proc. Natl. Acad. Sci. U S A 101, 11755–11760. 10.1073/pnas.0404432101 15284443 PMC511048

[B20] Chen J. F. Mandel E. M. Thomson J. M. Wu Q. Callis T. E. Hammond S. M.. (2006). The role of microRNA-1 and microRNA-133 in skeletal muscle proliferation and differentiation. Nat. Genet. 38, 228–233. 10.1038/ng1725 16380711 PMC2538576

[B21] Chhabra R. Adlakha Y. K. Hariharan M. Scaria V. Saini N. (2009). Upregulation of miR-23a-27a-24–2 cluster induces caspase-dependent and -independent apoptosis in human embryonic kidney cells. PLoS One 4:e5848. 10.1371/journal.pone.0005848 19513126 PMC2689653

[B22] Cobb B. S. Hertweck A. Smith J. O’Connor E. Graf D. Cook T.. (2006). A role for Dicer in immune regulation. J. Exp. Med. 203, 2519–2527. 10.1084/jem.20061692 17060477 PMC2118134

[B23] Cogswell J. P. Ward J. Taylor I. A. Waters M. Shi Y. Cannon B.. (2008). Identification of miRNA changes in Alzheimer’s disease brain and CSF yields putative biomarkers and insights into disease pathways. J. Alzheimers Dis. 14, 27–41. 18525125 10.3233/jad-2008-14103

[B24] Culpan D. Kehoe P. G. Love S. (2011). Tumour necrosis factor-alpha (TNF-alpha) and miRNA expression in frontal and temporal neocortex in Alzheimer’s disease and the effect of TNF-alpha on miRNA expression *in vitro*. Int. J. Mol. Epidemiol. Genet. 2, 156–162. 21686130 PMC3110390

[B25] Di Bona D. Plaia A. Vasto S. Cavallone L. Lescai F. Franceschi C.. (2008). Association between the interleukin-1beta polymorphisms and Alzheimer’s disease: a systematic review and meta-analysis. Brain Res. Rev. 59, 155–163. 10.1016/j.brainresrev.2008.07.003 18675847

[B26] Di Bona D. Vasto S. Capurso C. Christiansen L. Deiana L. Franceschi C.. (2009). Effect of interleukin-6 polymorphisms on human longevity: a systematic review and meta-analysis. Ageing Res. Rev. 8, 36–42. 10.1016/j.arr.2008.09.001 18930842

[B27] Dudda J. C. Salaun B. Ji Y. Palmer D. C. Monnot G. C. Merck E.. (2013). MicroRNA-155 is required for effector CD8+ T cell responses to virus infection and cancer. Immunity 38, 742–753. 10.1016/j.immuni.2012.12.006 23601686 PMC3788592

[B28] Ethell D. W. Shippy D. Cao C. Cracchiolo J. R. Runfeldt M. Blake B.. (2006). Abeta-specific T-cells reverse cognitive decline and synaptic loss in Alzheimer’s mice. Neurobiol. Dis. 23, 351–361. 10.1016/j.nbd.2006.03.008 16733088

[B29] Faraoni I. Antonetti F. R. Cardone J. Bonmassar E. (2009). miR-155 gene: a typical multifunctional microRNA. Biochim. Biophys. Acta 1792, 497–505. 10.1016/j.bbadis.2009.02.013 19268705

[B30] Filipowicz W. Jaskiewicz L. Kolb F. A. Pillai R. S. (2005). Post-transcriptional gene silencing by siRNAs and miRNAs. Curr. Opin. Struct. Biol. 15, 331–341. 10.1016/j.sbi.2005.05.006 15925505

[B31] Fillit H. Ding W. H. Buee L. Kalman J. Altstiel L. Lawlor B.. (1991). Elevated circulating tumor necrosis factor levels in Alzheimer’s disease. Neurosci. Lett. 129, 318–320. 10.1016/0304-3940(91)90490-k 1745413

[B32] Finch C. E. Morgan T. E. (2007). Systemic inflammation, infection, ApoE alleles and Alzheimer disease: a position paper. Curr. Alzheimer Res. 4, 185–189. 10.2174/156720507780362254 17430245

[B33] Fisher Y. Nemirovsky A. Baron R. Monsonego A. (2010). T cells specifically targeted to amyloid plaques enhance plaque clearance in a mouse model of Alzheimer’s disease. PLoS One 5:e10830. 10.1371/journal.pone.0010830 20520819 PMC2877087

[B34] Foldes-Papp Z. König K. Studier H. Bückle R. Breunig H. G. Uchugonova A.. (2009). Trafficking of mature miRNA-122 into the nucleus of live liver cells. Curr. Pharm. Biotechnol. 10, 569–578. 10.2174/138920109789069332 19619125

[B35] Galimberti D. Villa C. Fenoglio C. Serpente M. Ghezzi L. Cioffi S. M.. (2014). Circulating miRNAs as potential biomarkers in Alzheimer’s disease. J. Alzheimers Dis. 42, 1261–1267. 10.3233/JAD-140756 25024331

[B36] Gatto G. Rossi A. Rossi D. Kroening S. Bonatti S. Mallardo M. (2008). Epstein-Barr virus latent membrane protein 1 trans-activates miR-155 transcription through the NF-kappaB pathway. Nucleic Acids Res. 36, 6608–6619. 10.1093/nar/gkn666 18940871 PMC2582607

[B37] Georgantas R. W. 3rd Hildreth R. Morisot S. Alder J. Liu C. G. Heimfeld S.. (2007). CD34+ hematopoietic stem-progenitor cell microRNA expression and function: a circuit diagram of differentiation control. Proc. Natl. Acad. Sci. U S A 104, 2750–2755. 10.1073/pnas.0610983104 17293455 PMC1796783

[B38] Gracias D. T. Stelekati E. Hope J. L. Boesteanu A. C. Doering T. A. Norton J.. (2013). The microRNA miR-155 controls CD8(+) T cell responses by regulating interferon signaling. Nat. Immunol. 14, 593–602. 10.1038/ni.2576 23603793 PMC3664306

[B39] Gregory R. I. Shiekhattar R. (2005). MicroRNA biogenesis and cancer. Cancer Res. 65, 3509–3512. 10.1158/0008-5472.can-05-0298 15867338

[B40] Griffin W. S. Mrak R. E. (2002). Interleukin-1 in the genesis and progression of and risk for development of neuronal degeneration in Alzheimer’s disease. J. Leukoc. Biol. 72, 233–238. 12149413 PMC3835694

[B41] Guedes J. R. Custódia C. M. Silva R. J. de Almeida L. P. Pedroso de Lima M. C. Cardoso A. L. (2014). Early miR-155 upregulation contributes to neuroinflammation in Alzheimer’s disease triple transgenic mouse model. Hum. Mol. Genet. 23, 6286–6301. 10.1093/hmg/ddu348 24990149

[B42] Harrington L. E. Hatton R. D. Mangan P. R. Turner H. Murphy T. L. Murphy K. M.. (2005). Interleukin 17-producing CD4+ effector T cells develop via a lineage distinct from the T helper type 1 and 2 lineages. Nat. Immunol. 6, 1123–1132. 10.1038/ni1254 16200070

[B43] Hohlfeld R. Kerschensteiner M. Stadelmann C. Lassmann H. Wekerle H. (2006). The neuroprotective effect of inflammation: implications for the therapy of multiple sclerosis. Neurol. Sci. 27(Suppl. 1), S1–S7. 10.1007/s10072-006-0537-7 16708174

[B44] Huffaker T. B. Hu R. Runtsch M. C. Bake E. Chen X. Zhao J.. (2012). Epistasis between microRNAs 155 and 146a during T cell-mediated antitumor immunity. Cell Rep. 2, 1697–1709. 10.1016/j.celrep.2012.10.025 23200854 PMC3628775

[B45] Jindra P. T. Bagley J. Godwin J. G. Iacomini J. (2010). Costimulation-dependent expression of microRNA-214 increases the ability of T cells to proliferate by targeting Pten. J. Immunol. 185, 990–997. 10.4049/jimmunol.1000793 20548023 PMC3004219

[B46] Johnson C. D. Esquela-Kerscher A. Stefani G. Byrom M. Kelnar K. Ovcharenko D.. (2007). The let-7 microRNA represses cell proliferation pathways in human cells. Cancer Res. 67, 7713–7722. 10.1158/0008-5472.can-07-1083 17699775

[B47] Junker A. Krumbholz M. Eisele S. Mohan H. Augstein F. Bittner R.. (2009). MicroRNA profiling of multiple sclerosis lesions identifies modulators of the regulatory protein CD47. Brain 132, 3342–3352. 10.1093/brain/awp300 19952055

[B48] Ke Z. J. Bowen W. M. Gibson G. E. (2006). Peripheral inflammatory mechanisms modulate microglial activation in response to mild impairment of oxidative metabolism. Neurochem. Int. 49, 548–556. 10.1016/j.neuint.2006.04.011 16781017

[B49] Kim V. N. Nam J. W. (2006). Genomics of microRNA. Trends Genet. 22, 165–173. 10.1016/j.tig.2006.01.003 16446010

[B50] Kocerha J. Faghihi M. A. Lopez-Toledano M. A. Huang J. Ramsey A. J. Caron M. G.. (2009). MicroRNA-219 modulates NMDA receptor-mediated neurobehavioral dysfunction. Proc. Natl. Acad. Sci. U S A 106, 3507–3512. 10.1073/pnas.0805854106 19196972 PMC2651305

[B51] Kohlhaas S. Garden O. A. Scudamore C. Turner M. Okkenhaug K. Vigorito E. (2009). Cutting edge: the Foxp3 target miR-155 contributes to the development of regulatory T cells. J. Immunol. 182, 2578–2582. 10.4049/jimmunol.0803162 19234151

[B52] Kopp K. L. Ralfkiaer U. Gjerdrum L. M. Helvad R. Pedersen I. H. Litman T.. (2013). STAT5-mediated expression of oncogenic miR-155 in cutaneous T-cell lymphoma. Cell cycle 12, 1939–1947. 10.4161/cc.24987 23676217 PMC3735708

[B53] Korn T. Bettelli E. Gao W. Awasthi A. Jager A. Strom T. B.. (2007). IL-21 initiates an alternative pathway to induce proinflammatory T(H)17 cells. Nature 448, 484–487. 10.1038/nature05970 17581588 PMC3805028

[B54] Lafaille J. J. (1998). The role of helper T cell subsets in autoimmune diseases. Cytokine Growth Factor Rev. 9, 139–151. 10.1016/s1359-6101(98)00009-4 9754708

[B55] Lal H. Forster M. J. (1988). Autoimmunity and age-associated cognitive decline. Neurobiol. Aging 9, 733–742. 10.1016/s0197-4580(88)80141-6 3062479

[B56] Langrish C. L. Chen Y. Blumenschein W. M. Mattson J. Basham B. Sedgwick J. D.. (2005). IL-23 drives a pathogenic T cell population that induces autoimmune inflammation. J. Exp. Med. 201, 233–240. 10.1084/jem.20041257 15657292 PMC2212798

[B57] Larbi A. Pawelec G. Witkowski J. M. Schipper H. M. Derhovanessian E. Goldeck D.. (2009). Dramatic shifts in circulating CD4 but not CD8 T cell subsets in mild Alzheimer’s disease. J. Alzheimers Dis. 17, 91–103. 10.3233/JAD-2009-1015 19494434

[B58] Lawrie C. H. Soneji S. Marafioti T. Cooper C. D. Palazzo S. Paterson J. C.. (2007). MicroRNA expression distinguishes between germinal center B cell-like and activated B cell-like subtypes of diffuse large B cell lymphoma. Int. J. Cancer 121, 1156–1161. 10.1002/ijc.22800 17487835

[B59] Lee Y. S. Nakahara K. Pham J. W. Kim K. He Z. Sontheimer E. J.. (2004). Distinct roles for Drosophila Dicer-1 and Dicer-2 in the siRNA/miRNA silencing pathways. Cell 117, 69–81. 10.1016/S0092-8674(04)00261-2 15066283

[B60] Lind E. F. Elford A. R. Ohashi P. S. (2013). Micro-RNA 155 is required for optimal CD8+ T cell responses to acute viral and intracellular bacterial challenges. J. Immunol. 190, 1210–1216. 10.4049/jimmunol.1202700 23275599

[B61] Lombardi V. R. Fernández-Novoa L. Etcheverria I. Seoane S. Cacabelos R. (2004). Association between APOE epsilon4 allele and increased expression of CD95 on T cells from patients with Alzheimer’s disease. Methods Find. Exp. Clin. Pharmacol. 26, 523–529. 10.1358/mf.2004.26.7.863735 15538542

[B62] Lombardi V. R. Garcia M. Rey L. Cacabelos R. (1999). Characterization of cytokine production, screening of lymphocyte subset patterns and *in vitro* apoptosis in healthy and Alzheimer’s Disease (AD) individuals. J. Neuroimmunol. 97, 163–171. 10.1016/s0165-5728(99)00046-6 10408971

[B63] Lopez-Ramirez M. A. Wu D. Pryce G. Simpson J. E. Reijerkerk A. King-Robson J.. (2014). MicroRNA-155 negatively affects blood-brain barrier function during neuroinflammation. FASEB J. 28, 2551–2565. 10.1096/fj.13-248880 24604078

[B64] Lu L. F. Boldin M. P. Chaudhry A. Lin L. L. Taganov K. D. Hanada T.. (2010). Function of miR-146a in controlling Treg cell-mediated regulation of Th1 responses. Cell 142, 914–929. 10.1016/j.cell.2010.08.012 20850013 PMC3049116

[B65] Lu J. Getz G. Miska E. A. Alvarez-Saavedra E. Lamb J. Peck D.. (2005). MicroRNA expression profiles classify human cancers. Nature 435, 834–838. 10.1038/nature03702 15944708

[B66] Lu L. F. Thai T. H. Calado D. P. Chaudhry A. Kubo M. Tanaka K.. (2009). Foxp3-dependent microRNA155 confers competitive fitness to regulatory T cells by targeting SOCS1 protein. Immunity 30, 80–91. 10.1016/j.immuni.2008.11.010 19144316 PMC2654249

[B67] Maegdefessel L. (2014). The emerging role of microRNAs in cardiovascular disease. J. Intern. Med. 276, 633–644. 10.1111/joim.12298 25160930

[B68] Man S. M. Ma Y. R. Shang D. S. Zhao W. D. Li B. Guo D. W.. (2007). Peripheral T cells overexpress MIP-1alpha to enhance its transendothelial migration in Alzheimer’s disease. Neurobiol. Aging 28, 485–496. 10.1016/j.neurobiolaging.2006.02.013 16600437

[B69] Martin M. M. Lee E. J. Buckenberger J. A. Schmittgen T. D. Elton T. S. (2006). MicroRNA-155 regulates human angiotensin II type 1 receptor expression in fibroblasts. J. Biol. Chem. 281, 18277–18284. 10.1074/jbc.M601496200 16675453

[B70] Mastrangelo M. A. Sudol K. L. Narrow W. C. Bowers W. J. (2009). Interferon-gamma differentially affects Alzheimer’s disease pathologies and induces neurogenesis in triple transgenic-AD mice. Am. J. Pathol. 175, 2076–2088. 10.2353/ajpath.2009.090059 19808651 PMC2774071

[B71] Mattila K. M. Pirttilä T. Blennow K. Wallin A. Viitanen M. Frey H. (1994). Altered blood-brain-barrier function in Alzheimer’s disease? Acta Neurol. Scand. 89, 192–198. 10.1111/j.1600-0404.1994.tb01660.x 8030400

[B72] McGeer P. L. McGeer E. G. (2002). Innate immunity, local inflammation and degenerative disease. Sci. Aging Knowledge Environ. 2002:re3. 10.1126/sageke.2002.29.re3 14602998

[B73] McQuillan K. Lynch M. A. Mills K. H. (2010). Activation of mixed glia by Abeta-specific Th1 and Th17 cells and its regulation by Th2 cells. Brain Behav. Immun. 24, 598–607. 10.1016/j.bbi.2010.01.003 20060887

[B74] Miossec P. Korn T. Kuchroo V. K. (2009). Interleukin-17 and type 17 helper T cells. N. Engl. J. Med. 361, 888–898. 10.1056/NEJMra0707449 19710487

[B75] Monsonego A. Imitola J. Petrovic S. Zota V. Nemirovsky A. Baron R.. (2006). Abeta-induced meningoencephalitis is IFN-gamma-dependent and is associated with T cell-dependent clearance of Abeta in a mouse model of Alzheimer’s disease. Proc. Natl. Acad. Sci. U S A 103, 5048–5053. 10.1073/pnas.0506209103 16549802 PMC1458792

[B76] Monsonego A. Imitola J. Zota V. Oida T. Weiner H. L. (2003). Microglia-mediated nitric oxide cytotoxicity of T cells following amyloid beta-peptide presentation to Th1 cells. J. Immunol. 171, 2216–2224. 10.4049/jimmunol.171.5.2216 12928365

[B77] Murphy A. C. Lalor S. J. Lynch M. A. Mills K. H. (2010). Infiltration of Th1 and Th17 cells and activation of microglia in the CNS during the course of experimental autoimmune encephalomyelitis. Brain Behav. Immun. 24, 641–651. 10.1016/j.bbi.2010.01.014 20138983

[B78] Nagelkerken L. (1998). Role of Th1 and Th2 cells in autoimmune demyelinating disease. Braz. J. Med. Biol. Res. 31, 55–60. 10.1590/s0100-879x1998000100007 9686179

[B79] Neilson J. R. Zheng G. X. Burge C. B. Sharp P. A. (2007). Dynamic regulation of miRNA expression in ordered stages of cellular development. Genes Dev. 21, 578–589. 10.1101/gad.1522907 17344418 PMC1820899

[B80] Nelson P. T. Keller J. N. (2007). RNA in brain disease: no longer just “the messenger in the middle”. J. Neuropathol. Exp. Neurol. 66, 461–468. 10.1097/01.jnen.0000240474.27791.f3 17549006

[B81] Nelson P. T. Wang W. X. Rajeev B. W. (2008). MicroRNAs (miRNAs) in neurodegenerative diseases. Brain Pathol. 18, 130–138. 10.1111/j.1750-3639.2007.00120.x 18226108 PMC2859437

[B82] O’Connell R. M. Chaudhuri A. A. Rao D. S. Baltimore D. (2009). Inositol phosphatase SHIP1 is a primary target of miR-155. Proc. Natl. Acad. Sci. U S A 106, 7113–7118. 10.1073/pnas.0902636106 19359473 PMC2678424

[B83] O’Connell R. M. Kahn D. Gibson W. S. Round J. L. Scholz R. L. Chaudhuri A. A.. (2010). MicroRNA-155 promotes autoimmune inflammation by enhancing inflammatory T cell development. Immunity 33, 607–619. 10.1016/j.immuni.2010.09.009 20888269 PMC2966521

[B84] O’Connell R. M. Taganov K. D. Boldin M. P. Cheng G. Baltimore D. (2007). MicroRNA-155 is induced during the macrophage inflammatory response. Proc. Natl. Acad. Sci. U S A 104, 1604–1609. 10.1073/pnas.0610731104 17242365 PMC1780072

[B85] Park C. Y. Choi Y. S. McManus M. T. (2010). Analysis of microRNA knockouts in mice. Hum. Mol. Genet. 19, R169–R175. 10.1093/hmg/ddq367 20805106 PMC2981466

[B86] Pellicano M. Larbi A. Goldeck D. Colonna-Romano G. Buffa S. Bulati M.. (2012). Immune profiling of Alzheimer patients. J. Neuroimmunol. 242, 52–59. 10.1016/j.jneuroim.2011.11.005 22153977

[B87] Remarque E. J. Bollen E. L. Weverling-Rijnsburger A. W. Laterveer J. C. Blauw G. J. Westendorp R. G. (2001). Patients with Alzheimer’s disease display a pro-inflammatory phenotype. Exp. Gerontol. 36, 171–176. 10.1016/s0531-5565(00)00176-5 11162920

[B88] Richartz-Salzburger E. Batra A. Stransky E. Laske C. Köhler N. Bartels M.. (2007). Altered lymphocyte distribution in Alzheimer’s disease. J. Psychiatr. Res. 41, 174–178. 10.1016/j.jpsychires.2006.01.010 16516234

[B89] Rodriguez A. Vigorito E. Clare S. Warren M. V. Couttet P. Soond D. R.. (2007). Requirement of bic/microRNA-155 for normal immune function. Science 316, 608–611. 10.1126/science.1139253 17463290 PMC2610435

[B90] Romero P. Zippelius A. Kurth I. Pittet M. J. Touvrey C. Iancu E. M.. (2007). Four functionally distinct populations of human effector-memory CD8+ T lymphocytes. J. Immunol. 178, 4112–4119. 10.4049/jimmunol.178.7.4112 17371966

[B91] Rouas R. Fayyad-Kazan H. El Zein N. Lewalle P. Rothé F. Simion A.. (2009). Human natural Treg microRNA signature: role of microRNA-31 and microRNA-21 in FOXP3 expression. Eur. J. Immunol. 39, 1608–1618. 10.1002/eji.200838509 19408243

[B92] Rubio-Perez J. M. Morillas-Ruiz J. M. (2012). A review: inflammatory process in Alzheimer’s disease, role of cytokines. ScientificWorldJournal 2012:756357. 10.1100/2012/756357 22566778 PMC3330269

[B93] Rusca N. Dehò L. Montagner S. Zielinski C. E. Sica A. Sallusto F.. (2012). MiR-146a and NF-kappaB1 regulate mast cell survival and T lymphocyte differentiation. Mol. Cell. Biol. 32, 4432–4444. 10.1128/mcb.00824-12 22927641 PMC3486148

[B94] Salaun B. Yamamoto T. Badran B. Tsunetsugu-Yokota Y. Roux A. Baitsch L.. (2011). Differentiation associated regulation of microRNA expression *in vivo* in human CD8+ T cell subsets. J. Transl. Med. 9:44. 10.1186/1479-5876-9-44 21507256 PMC3098162

[B95] Sardi F. Fassina L. Venturini L. Inguscio M. Guerriero F. Rolfo E.. (2011). Alzheimer’s disease, autoimmunity and inflammation. The good, the bad and the ugly. Autoimmun. Rev. 11, 149–153. 10.1016/j.autrev.2011.09.005 21996556

[B96] Saresella M. Calabrese E. Marventano I. Piancone F. Gatti A. Alberoni M.. (2011). Increased activity of Th-17 and Th-9 lymphocytes and a skewing of the post-thymic differentiation pathway are seen in Alzheimer’s disease. Brain Behav. Immun. 25, 539–547. 10.1016/j.bbi.2010.12.004 21167930

[B97] Schindowski K. Eckert A. Peters J. Gorriz C. Schramm U. Weinandi T.. (2007). Increased T-cell reactivity and elevated levels of CD8+ memory T-cells in Alzheimer’s disease-patients and T-cell hyporeactivity in an Alzheimer’s disease-mouse model: implications for immunotherapy. Neuromolecular Med. 9, 340–354. 10.1007/s12017-007-8015-9 17963048

[B98] Schultz J. Lorenz P. Gross G. Ibrahim S. Kunz M. (2008). MicroRNA let-7b targets important cell cycle molecules in malignant melanoma cells and interferes with anchorage-independent growth. Cell Res. 18, 549–557. 10.1038/cr.2008.45 18379589

[B99] Skinner J. P. Keown A. A. Chong M. M. (2014). The miR-17 approximately 92a cluster of microRNAs is required for the fitness of Foxp3+ regulatory T cells. PLoS One 9:e88997. 10.1371/journal.pone.0088997 24523948 PMC3921252

[B100] Smigielska-Czepiel K. van den Berg A. Jellema P. van der Lei R. J. Bijzet J. Kluiver J.. (2014). Comprehensive analysis of miRNA expression in T-cell subsets of rheumatoid arthritis patients reveals defined signatures of naive and memory Tregs. Genes Immun. 15, 115–125. 10.1038/gene.2013.69 24401767 PMC3959220

[B101] Sonkoly E. Janson P. Majuri M. L. Savinko T. Fyhrquist N. Eidsmo L.. (2010). MiR-155 is overexpressed in patients with atopic dermatitis and modulates T-cell proliferative responses by targeting cytotoxic T lymphocyte-associated antigen 4. J. Allergy Clin. Immunol. 126, 581.e1–589.e20. 10.1016/j.jaci.2010.05.045 20673989

[B102] Speciale L. Calabrese E. Saresella M. Tinelli C. Mariani C. Sanvito L.. (2007). Lymphocyte subset patterns and cytokine production in Alzheimer’s disease patients. Neurobiol. Aging 28, 1163–1169. 10.1016/j.neurobiolaging.2006.05.020 16814429

[B103] Starr T. K. Jameson S. C. Hogquist K. A. (2003). Positive and negative selection of T cells. Annu. Rev. Immunol. 21, 139–176. 10.1146/annurev.immunol.21.120601.141107 12414722

[B104] Steinman L. (2006). State of the art. Four easy pieces: interconnections between tissue injury, intermediary metabolism, autoimmunity and chronic degeneration. Proc. Am. Thorac. Soc. 3, 484–486. 10.1513/pats.200603-061ms 16921119

[B105] Strauss S. Bauer J. Ganter U. Jonas U. Berger M. Volk B. (1992). Detection of interleukin-6 and alpha 2-macroglobulin immunoreactivity in cortex and hippocampus of Alzheimer’s disease patients. Lab. Invest. 66, 223–230. 1370967

[B106] Taganov K. D. Boldin M. P. Chang K. J. Baltimore D. (2006). NF-kappaB-dependent induction of microRNA miR-146, an inhibitor targeted to signaling proteins of innate immune responses. Proc. Natl. Acad. Sci. U S A 103, 12481–12486. 10.1073/pnas.0605298103 16885212 PMC1567904

[B107] Tam W. (2001). Identification and characterization of human BIC, a gene on chromosome 21 that encodes a noncoding RNA. Gene 274, 157–167. 10.1016/s0378-1119(01)00612-6 11675008

[B108] Tang Q. Bluestone J. A. (2006). Regulatory T-cell physiology and application to treat autoimmunity. Immunol. Rev. 212, 217–237. 10.1111/j.0105-2896.2006.00421.x 16903917

[B109] Tarasenko T. Kole H. K. Chi A. W. Mentink-Kane M. M. Wynn T. A. Bolland S. (2007). T cell-specific deletion of the inositol phosphatase SHIP reveals its role in regulating Th1/Th2 and cytotoxic responses. Proc. Natl. Acad. Sci. U S A 104, 11382–11387. 10.1073/pnas.0704853104 17585010 PMC2040907

[B110] Tarkowski E. Wallin A. Regland B. Blennow K. Tarkowski A. (2001). Local and systemic GM-CSF increase in Alzheimer’s disease and vascular dementia. Acta Neurol. Scand. 103, 166–174. 10.1034/j.1600-0404.2001.103003166.x 11240564

[B111] Thai T. H. Calado D. P. Casola S. Ansel K. M. Xiao C. Xue Y.. (2007). Regulation of the germinal center response by microRNA-155. Science 316, 604–608. 10.1126/science.1141229 17463289

[B112] Tili E. Michaille J. J. Cimino A. Costinean S. Dumitru C. D. Adair B.. (2007). Modulation of miR-155 and miR-125b levels following lipopolysaccharide/TNF-alpha stimulation and their possible roles in regulating the response to endotoxin shock. J. Immunol. 179, 5082–5089. 10.4049/jimmunol.179.8.5082 17911593

[B113] Togo T. Akiyama H. Iseki E. Kondo H. Ikeda K. Kato M.. (2002). Occurrence of T cells in the brain of Alzheimer’s disease and other neurological diseases. J. Neuroimmunol. 124, 83–92. 10.1016/s0165-5728(01)00496-9 11958825

[B114] Town T. Tan J. Flavell R. A. Mullan M. (2005). T-cells in Alzheimer’s disease. Neuromolecular Med. 7, 255–264. 10.1385/NMM:7:3:255 16247185

[B115] Volinia S. Calin G. A. Liu C. G. Ambs S. Cimmino A. Petrocca F.. (2006). A microRNA expression signature of human solid tumors defines cancer gene targets. Proc. Natl. Acad. Sci. U S A 103, 2257–2261. 10.1073/pnas.0510565103 16461460 PMC1413718

[B116] Vugler A. Lawrence J. Walsh J. Carr A. Gias C. Semo M.. (2007). Embryonic stem cells and retinal repair. Mech. Dev. 124, 807–829. 10.1016/j.mod.2007.08.002 17881192

[B117] Weiner H. L. Lemere C. A. Maron R. Spooner E. T. Grenfell T. J. Mori C.. (2000). Nasal administration of amyloid-beta peptide decreases cerebral amyloid burden in a mouse model of Alzheimer’s disease. Ann. Neurol. 48, 567–579. 10.1002/1531-8249(200010)48:4<567::aid-ana3>3.3.co;2-n 11026440

[B118] Wimo A. Jonsson L. Winblad B. (2006). An estimate of the worldwide prevalence and direct costs of dementia in 2003. Dement. Geriatr. Cogn. Disord. 21, 175–181. 10.1159/000090733 16401889

[B119] Wolf S. A. Steiner B. Akpinarli A. Kammertoens T. Nassenstein C. Braun A.. (2009). CD4-positive T lymphocytes provide a neuroimmunological link in the control of adult hippocampal neurogenesis. J. Immunol. 182, 3979–3984. 10.4049/jimmunol.0801218 19299695

[B120] Wyss-Coray T. Lin C. Yan F. Yu G. Q. Rohde M. McConlogue L.. (2001). TGF-beta1 promotes microglial amyloid-beta clearance and reduces plaque burden in transgenic mice. Nat. Med. 7, 612–618. 10.1038/87945 11329064

[B121] Xu W. Li J. Y. (2007). MicroRNA gene expression in malignant lymphoproliferative disorders. Chin. Med. J. (Engl) 120, 996–999. 17624268

[B122] Xue S. R. Xu D. H. Yang X. X. Dong W. L. (2009). Alterations in lymphocyte subset patterns and co-stimulatory molecules in patients with Alzheimer disease. Chin. Med. J. (Engl) 122, 1469–1472. 19567174

[B123] Yang L. Boldin M. P. Yu Y. Liu C. S. Ea C. K. Ramakrishnan P.. (2012). miR-146a controls the resolution of T cell responses in mice. J. Exp. Med. 209, 1655–1670. 10.1084/jem.20112218 22891274 PMC3428948

[B124] Yin Q. McBride J. Fewell C. Lacey M. Wang X. Lin Z.. (2008). MicroRNA-155 is an Epstein-Barr virus-induced gene that modulates Epstein-Barr virus-regulated gene expression pathways. J. Virol. 82, 5295–5306. 10.1128/jvi.02380-07 18367535 PMC2395216

[B125] Yu Y. Chai J. Zhang H. Chu W. Liu L. Ma L.. (2014). miR-194 promotes burn-induced hyperglycemia via attenuating IGF-IR expression. Shock 42, 578–584. 10.1097/SHK.0000000000000258 25186839

[B126] Zhang N. Bevan M. J. (2010). Dicer controls CD8+ T-cell activation, migration and survival. Proc. Natl. Acad. Sci. U S A 107, 21629–21634. 10.1073/pnas.1016299107 21098294 PMC3003005

